# What Does Cytochrome Oxidase Histochemistry Represent in the Visual Cortex?

**DOI:** 10.3389/fnana.2016.00079

**Published:** 2016-07-20

**Authors:** Toru Takahata

**Affiliations:** Laboratory of Comparative Molecular Neuroanatomy, Interdisciplinary Institute of Neuroscience and Technology, Zhejiang UniversityHangzhou, China

**Keywords:** *c-fos*, activity dependent, ocular dominance columns, CO blobs/puffs/patches, CO stripes, vesicular glutamate transporter 2

## Background

Ever since Wong–Riley first reported in the late 1970s that histological staining using the chemical reactions of cytochrome oxidase (CO), a metabolic enzyme in the mitochondria, is useful to reveal the cytoarchitecture of the brain (Wong-Riley, 1979), the CO histochemistry method has been widely used in the field of neuroanatomy, especially in carnivores and primates. It has been suggested that CO activity is coupled with the spike activity of neurons (Wong-Riley, 1979). Most strikingly, the use of CO histochemistry was critical to the discovery of patchy functional sub-compartments in the supragranular layers of primate V1, which are referred to as “CO blobs/puffs/patches” (Horton, 1984). Additionally, CO histochemistry revealed sub-compartments of “thick stripes,” “thin stripes,” and “pale stripes” in the middle layer of the secondary visual cortex (V2), which have been shown to possess distinct connections with V1 and other cortical areas (Sincich and Horton, 2002). These three stripes are also functionally distinct, as binocular disparity coding neurons are clustered into thick stripes (Chen et al., 2008). As such, CO histochemistry has revealed many normally cryptic functional compartments of the mammalian brain. Nonetheless, in this article, we aim to question the interpretation of results from CO histochemistry as “activity maps” of the brain.

## Inconsistencies with immediate-early gene (IEG) expression

Immediate-early genes (IEGs), such as *c-FOS, ZIF268/EGR-1*, and *BDNF*, are quickly transcribed in response to the elevation of cytosolic calcium after post-synaptic activation, and their mRNA is quickly degraded after the offset of neuronal activity (Zangenehpour and Chaudhuri, 2002). Thus, IEG histochemistry is also often used for the activity mapping of neurons. We observed, however, that CO staining patterns do not correspond to the expression patterns of IEGs. Although layer 4 of the macaque V1 shows higher IEG expression similar to CO histochemistry, no evidence has been presented that IEGs are strongly expressed in V1 blobs or V2 stripes (Takahata et al., 2008). On the other hand, the expression of IEGs is generally stronger in layers 2, 3a, 3b, 4, and 6 than that in layers 3c (Brodmann's layer 4B) and 5 in the macaque V1, whereas CO staining densities are only high in blobs, layers 3Bβ (Brodmann's layer 4A) and layer 4 (Brodmann's layer 4C). In cases of monocular inactivation, we have shown that IEG changes are much faster than CO changes (Takahata et al., 2009a). Moreover, in owl monkeys, CO histochemistry failed to reveal ocular dominance columns (ODCs), whereas IEG expression clearly exhibited ODC stripe patterns (Takahata et al., 2014). This inconsistency was also discussed previously comparing differences between CO histochemistry and radiography studies of 2-deoxyglucose (2-DG) uptake, which is another histological technique designed to reveal activity maps (Wong-Riley, 1989). Mitochondrial metabolic changes are likely an indirect effect of changes in the spike activity of neurons. The IEG expression patterns are rather similar to 2-DG histochemistry.

## Consistencies with VGLUT2-immunoreactivity

Unlike IEG expressions, CO histochemistry shows a striking similarity to the immunoreactivity (ir) of vesicular glutamate transporter 2 (VGLUT2). *VGLUT2* mRNA is abundantly expressed in the excitatory projection neurons of the thalamus, but few are found in the cortex (Balaram et al., 2013). Because the gene product of *VGLUT2* is transferred to the axons to mediate glutamate trafficking, thalamo-cortical afferent terminals are visualized with little background when the cortical sections are stained with antibodies against VGLUT2 (Nakamura et al., 2007). Therefore, the input layers of layer 3Bβ and layer 4 of V1, layer 4 of the primary auditory cortex (A1), and the somatosensory cortex (S1) are densely labeled by VGLUT2 immunohistochemistry (IHC; Hackett and de la Mothe, 2009; Balaram et al., 2013). Interestingly, CO blobs in V1 and CO stripes in V2 have been shown to receive direct input from the koniocellular layers of LGN and the pulvinar nuclei, respectively (Livingstone and Hubel, 1982; Levitt et al., 1995; Ding and Casagrande, 1997), and they are also labeled by VGLUT2 IHC (Wong and Kaas, 2010; Garcia-Marin et al., 2013; Rockoff et al., 2014). Furthermore, CO histochemistry reveals a conspicuous reticular honey-comb structure in layer 3Bβ of V1, as does VGLUT2 IHC (Garcia-Marin et al., 2013). Thus, the staining patterns are quite similar between CO and VGLUT2-ir. This is consistent with species other than primates, such as tree shrews, opossums, and rats (Nakamura et al., 2007; Wong and Kaas, 2009; Balaram et al., 2015). These observations led us to consider the possibility that CO histochemistry labels almost the same matter as VGLUT2 IHC, which is the thalamo-cortical afferent terminal axons.

## Fibers vs. somata

**Figure 1:**
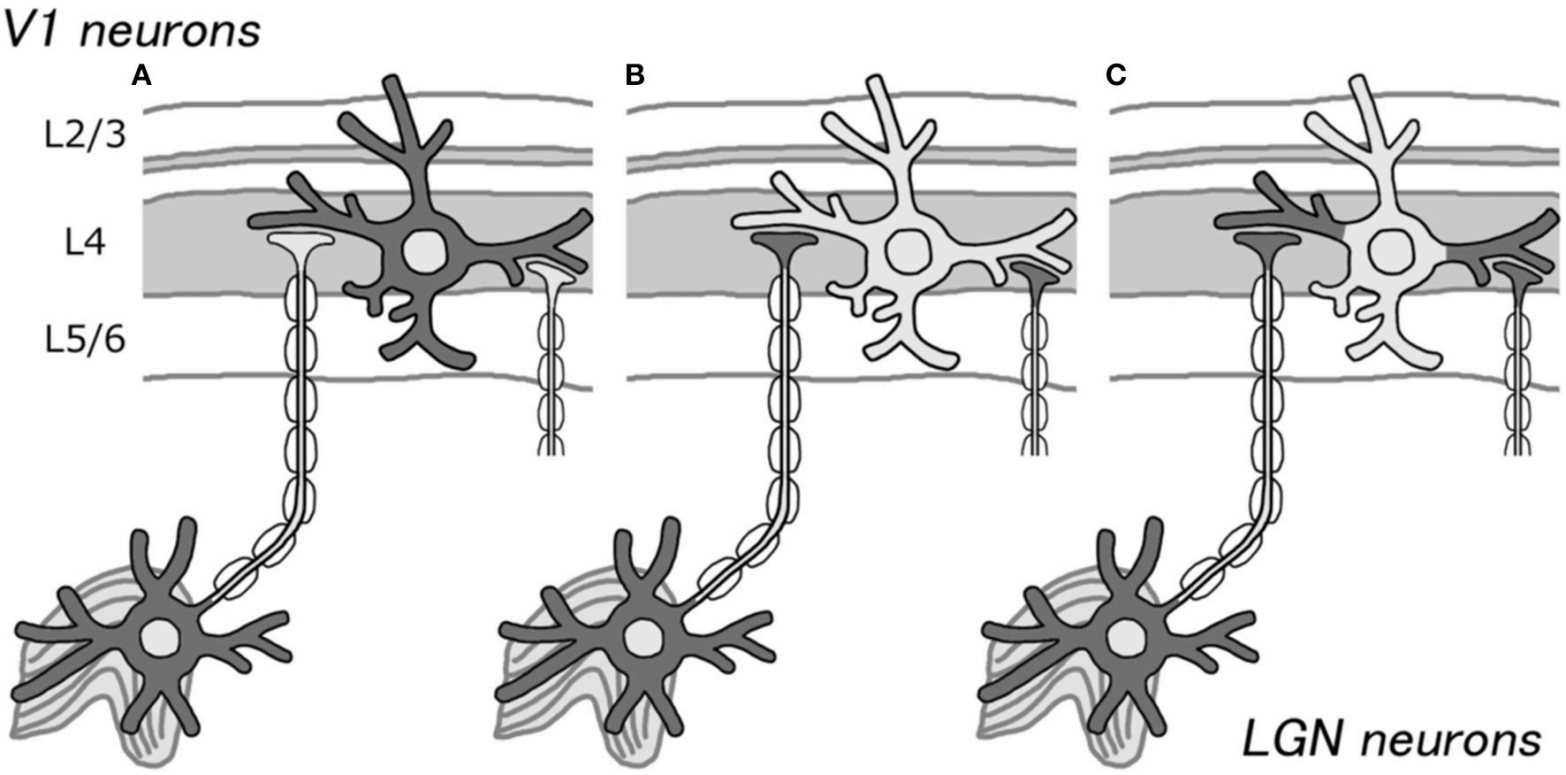
**Schematic illustrations for the source of CO activity in V1**. Previously, CO histochemistry was thought to represent the metabolic or spiking activity of cortical neurons **(A)**. We propose that it may represent thalamo-cortical afferent terminals, due to the presence of higher CO activity in LGN neurons than that in cortical neurons **(B)**. As another possibility, it may represent cortical dendrites that receive direct inputs from the LGN **(C)**. In either case, CO histochemistry probably does not reflect the activity of the somata of cortical neurons.

Bunches of fibers are visible when cortical sections stained for CO are closely observed using high magnification (Horton, 1984), but only a few somata show strong CO activity. This observation supports our idea that CO histochemistry, in fact, labels axons from the thalamus, but not highly active cortical neurons (Figures 1A,B). However, according to Carroll and Wong-Riley (1984), dendrites are the main source of CO activity, not axons. They used electron microscopy to show that most of the mitochondria reside in the post synaptic dendrites. Perhaps, these dendrites that directly contact the thalamo-cortical axons show intense CO activity, but the electrical activity does not reach to the somata due to inhibitory inputs onto the proximal parts of dendrites (Figure 1C). Previous neuroanatomical and intracellular recording studies showed that there are many transformations between thalamo-cortical afferents and their target dendrites (Kisvarday et al., 1986), and they are similar in their spiking activities (Jin et al., 2008; Priebe and Ferster, 2012), while not all dendritic spikes propagate into somata (Mainen et al., 1995), which supports our hypothesis. Another possibility is that the authors examined only cortico-cortical axons, not thalamo-cortical axons, and concluded that CO activity only resides in dendrites, ignoring the thalamo-cortical axons. The honey-comb structure of layer 3Bβ in V1 is obviously caused by axons from the thalamus, but it shows strong CO activity (Garcia-Marin et al., 2013).

Reportedly, CO activity patterns are slightly different from the tracer signals in layer 4 of V1 after monocular deprivation (Horton and Hocking, 1998). The relatively dense CO bands, which are supposed to represent high activity of the open eye, were narrower than the pale CO bands, which are supposed to represent low activity of the closed eye, while tracer signals showed comparable widths for both ODCs. Authors discuss that this discrepancy may be due to the decrease of neuronal activity in binocular border strip zones along the borders of ODCs. Our previous study also suggested that border strips have distinct circuit related to binocular lateral inhibition (Takahata et al., 2009a). We suggest, however, that this discrepancy results from different staining properties of the tritium tracer, which mostly reveals cell bodies of cortical recipient neurons, and CO, which mostly reveals distribution of active geniculate afferent fibers. It remains unknown why CO bands for the intact eye were narrower than the ones for the closed eye.

## Monocular deprivation treatment

People previously believed that CO histochemistry marked the activity maps of neurons primarily because of the observation that CO staining density significantly decreases in V1 after monocular enucleation or inactivation treatment (Wong-Riley, 1979). This interpretation, however, poses a question to us. We have realized that the mRNA expression levels of numerous genes significantly decrease in the LGN and V1 following monocular inactivation (Higo et al., 2000, 2002; Takahata et al., 2009b, 2010; Watakabe et al., 2009). Not only gene transcription, but also protein trafficking is down-regulated for PV and calbindin D-28K (CB) in the LGN and V1 (Blumcke et al., 1994). Even cell volumes show a significant decrease in the LGN (Hendrickson and Tigges, 1985). It appears that overall cellular activity is slowed down, especially in the neurons of the LGN after monocular inactivation treatment. Therefore, the metabolic activity of LGN neurons must be decreased as well, and therefore CO activity of thalamo-cortical afferent axons decreases after that. In other words, CO activity and neuronal activity of cortical neurons are not tightly and directly coupled. Only when severe damage (e.g., monocular enucleation) occurs in the sensory system, the reduction of CO is obvious. When the deprivation is not as strong (e.g., monocular eyelid suture), it is hard to see such an apparent reduction in CO activity in the brain.

## Re-interpretation of CO staining data

We agree that CO activity is coupled with neuronal activity in a broad sense, but this should not be the fundamental interpretation of CO histochemistry data. We suggest that the CO activity in thalamic neurons is generally higher than in cortical neurons, and the difference in CO activity among cortical neurons is likely smaller than the difference between thalamic neurons and cortical neurons. Thus, when thalamic neurons extend their axons into the cortex, their distribution is noticeable rather than the difference of activity among cortical neurons.

While cortical neuronal activity and thalamic input are closely linked, this distinction is not trivial, and changes, we presume, the previous interpretations of cortical functional organization. In contrast to models where functional organization is established based largely on relative weights and competition between intracortical connections, our interpretation gives substantial weight to the view that, in primary sensory areas, thalamo-cortical connectivity is the primary determinant of the fundamental nature of cortical compartments. For example, CO blobs in V1 should be defined as “compartments where LGN koniocellular projections terminate,” rather than “compartments where neurons are more active” or “compartments where color vision is processed and orientation preference does not exist (Livingstone and Hubel, 1984).” Similarly, CO thick and thin stripes in V2 should be defined as “compartments where afferents from the pulvinar nuclei terminate,” rather than “compartments where V1 blob neurons project.” Cortical neurons within blobs or thick/thin stripes are not necessarily more active than neurons outside of these compartments. Our new interpretation fits in nicely with novel ideas about origins of cortical columnar architecture suggested by several groups. According to them, the topographic maps of V1, such as orientation preference, ocular dominance and ON/OFF luminance polarity, are all generated onto the topographically arranged scaffolds provided by geniculate afferents into the cortex, rather than cortico-cortical interactions (Vidyasagar and Eysel, 2015; Kremkow et al., 2016; Lee et al., 2016). Similarly, although the origins of V2 stripe architecture are not well studied, pulvinar-cortical projections may play more crucial roles than cortico-cortical projections for the topographic organization of V2.

This interpretation change could also influence observations in future experiments. For example, if the primary motor cortex (M1) becomes dense with CO activity in a certain transgenic animal, it should be considered as revealing that M1 acquired projections from the thalamus in this animal, rather than revealing that M1 became electrically more active. In light of these considerations above, we also mention that CO histochemistry is a convenient histological method to use to reveal thalamo-cortical afferent terminal patterns, as it is easier to conduct compared to tracer studies or VGLUT2 IHC.

## Author contributions

The author confirms being the sole contributor of this work and approved it for publication.

## Funding

This publication was supported by the Key Construction Program of the National “985” Project, China, to TT.

### Conflict of interest statement

The author declares that the research was conducted in the absence of any commercial or financial relationships that could be construed as a potential conflict of interest.
